# Elderly people and responses to COVID-19 in 27 Countries

**DOI:** 10.1371/journal.pone.0235590

**Published:** 2020-07-02

**Authors:** J. -F. Daoust

**Affiliations:** 1 School of Social and Political Science, Politics and International Relations, University of Edinburgh, Edinburgh, Scotland, United Kingdom; 2 Center for the Study of Democratic Citizenship, Montreal, Quebec, Canada; Chinese Academy of Medical Sciences and Peking Union Medical College, CHINA

## Abstract

Amongst the most robust consensus related to the COVID-19 disease is that the elderly are by far the most vulnerable population group. Hence, public authorities target older people in order to convince them to comply with preventive measures. However, we still know little about older people’s attitudes and compliance toward these measures. In this research, I aim to improve our understanding of elderly people’s responses to the pandemic using data from 27 countries. Results are surprising and quite troubling. Elderly people’s response is substantially similar to their fellow citizens in their 50’s and 60’s. This research (i) provides the first thorough description of the most vulnerable population’s attitudes and compliance in a comparative perspective (ii) suggest that governments’ strategies toward elderly people are far from successful and (iii) shows that methodologically, we should be more cautious in treating age as having a linear effect on COVID-19 related outcomes.

## Introduction

The COVID-19 pandemic has forced governments to enact drastic public health measures in order to minimize the impact of the disease. These include social distancing, but also clear recommendations of lockdown for the elderly. That said, if my grandmother was alive, it would have been very hard to convince her not to go out and play cards at the social club even if the head of the government (that she voted for!) was urging elderlies to stay home. Beyond my anecdotical grandmother, we have seen many headlines in the media about generational differences. *The Boston Globe* titled an article “Call Your Mom: The Generational Politics of Covid-19” and *The Telegraph* wrote “‘Generation Me’ Must Start Thinking About Others if We’re to Stop the Spread of Coronavirus” as a headline. Moreover, on top of the media coverage, there is a burgeoning number of data on age differences in attitudes and compliance to preventive measures in the context of the Covid-19.

Epidemiologists are crystal clear: age is the most important factor in diminishing one’s chances to survive the COVID-19, especially after 65 years of age. [1–2] Hence, governments strategy around the world has notably focused on targeting elderly people and trying to convince them to comply with the public health preventive measures. [3] Given the well-known greater mortality rate among elderly people and the clear objective of the governments around the world, it is very reasonable to expect the elderly people to be the most dutiful group in the population. That is, we should expect them to be more willing to isolate if they were told to do so, and to comply with preventive measures to a greater extent than their younger fellow citizens.

In this research, I test these expectations using the Imperial College London and YouGov dataset. It is, to my knowledge, the most extensive dataset including attitudes toward COVID-19 and self-reported compliance to preventive measures. The results from 27 countries are unfortunately clear and quite troubling. That is, elderly people’s prospect to self-isolate and willingness to do so is *not* the greatest among the whole population. It is very similar to people in their 50’s and 60’s, despite the fact that they are much more likely to die from the disease. Moreover, the same applies to their level of compliance with many different COVID-19 preventive measures.

This research improves our understanding and response to the COVID-19 pandemic with clear descriptions, methodological implications and normative concerns. (i) It provides the first thorough description of the most vulnerable population’s attitudes and compliance to COVID-19 preventive measures in a comparative perspective. (ii) The results clearly suggest that scholars interested in the COVID-19 pandemic should not take for granted the linearity of the effect of age. (ii) It clearly shows that governments’ strategies are not meeting their objectives and thus should be revisited. The pandemic is far from over and we need to find better ways to minimize the number of deaths. While improving our understanding of elderly people is not a panacea, it would help minimize the number of deaths.

## Age is *not* just a number

Although we should be very cautious in dichotomizing age when it comes to deciding who needs intensive care by giving the resources to young people and not to the elderly (for an important discussion on the ethical considerations, see Monter-Odasso et al. [4]), age is clearly the most important factor in predicting the odds of surviving the COVID-19 disease. [1–2] In fact, this is among the most robust consensus among scientists. In an ideal world, that alone should justify the expectation that older people will be more dutiful in terms of following public health recommendations. However, the issue is not that straightforward.

COVID-19 is not the only disease for which the consequences are known to be the worst among older people. This is the case for invasive pneumococcal disease or heat stroke, among others. In both cases, there are existing preventive measures. However, Schneeberg et al. [5] have shown that, among Canadians, the relationship between age and having gotten a pneumococcal vaccine is non-linear. 45% of 65-69-year-olds did so, a proportion that increases to 67% among the 70-79-year-olds but decreases by 6 percentage points for the 80+ year-old group. [5] Moreover, Khare et al. [6] demonstrated that, among English people (where there was an important heat wave in 2013), older people were not more likely to have greater scores on “always keeping out of the sun between 100 and 1500.” The 26-60-year-olds were more likely to comply with that preventive behaviour, but the 76+ year-olds were not (compared to the youngest 18–25 group).

That said, the mortality rate of COVID-19 among elderly people is, objectively speaking, much greater than other diseases such as pneumococcal disease or heat stroke. Moreover, the governments' and citizens’ reactions and measures to minimize the consequences of that disease are of a totally different scale. Hence, it is very reasonable to expect that older people will be more dutiful when it comes to following the recommendation of public health agencies and governments.

This is what one of the first studies in the United States showed: older Americans were more likely to perceive COVID-19 as a “significant crisis” and as a threat to people’s health. [7] The effect was quite linear in both cases: the older the respondents, the more likely they were to perceive the COVID-19 as a threat. Several academic studies also examined the impact of age on attitudes toward the COVID-19 pandemic and compliance with different preventive measures. Many studies measure the effect of age using linear models, which is consistent with the raw findings of the Pew Research Center cited above and is, in fact, very intuitive–see, among others, Painter and Qiu [8] for a study on the US, Pfattheicher et al. [9] for a focus on the United Kingdom and Germany, and Brouard et al. [10] for France. Some do allow non-linear relationships between age and COVID-19 related attitudes or behaviours, but they only do so as a control (i.e. they focus other explanatory factors and thus only include age to obtain more precise estimates) and do not discuss its implications. [11–12] In other words, the majority of research does not include age as having a potentially non-linear effect on different variables related to COVID-19.

However, two studies provide more information on age and elderlies’ response to COVID-19. First, Barari et al. [13] do not focus on age but display their descriptive statistics over four different age groups, including those over 60 years old. Although, the authors do not discuss thoroughly the age effects, it appears that the 60+ age group is the most disciplined (or ‘dutiful’ group) in regards to all nine attitudes or measures of compliance towards preventive rules and procedures–see their Figs 1 and 4. Thus, Barari et al. [13] seem to confirm the expected relationship: citizens are more dutiful with age–at least in their sample of Italian people.

**Fig 1:**
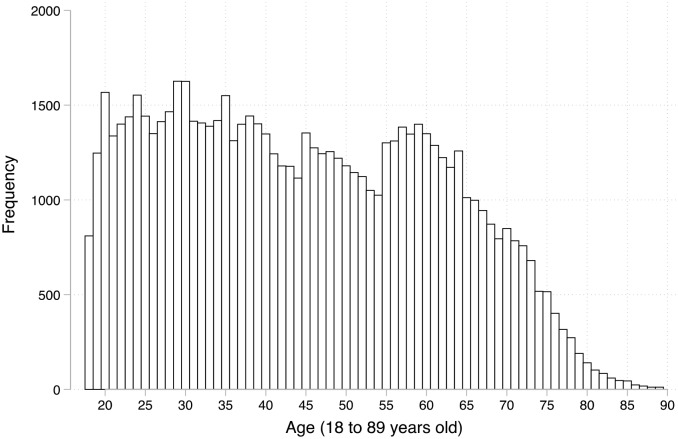
Age, prospective isolation, and willingness to isolate. Local regression with a kernel (epanechnikov) function and a bandwidth of 0.8, with 84% confidence intervals included. [19].

Another very insightful research is that of Canning et al. [14] who study a sample of US respondents. The authors allow non-linearity in their analyses by dividing age in six different groups–see in particular their Fig 3. Their findings not only show a non-linear relationship among age groups, but also that age has a contrary effect on social distancing than expected during the COVID-19 pandemic. This research, while insightful, is limited to a US sample, includes a low number of observations among certain age groups (like the >70 years of age) and focuses on social distancing. In this research, I aim to extend the sample to include 27 countries, with much more observations in each age group, and study more than just social distancing. Below, I detail the empirical strategy to do so.

## Data and indicators

### Data

The Institute of Global Health Innovation (IGHI) at Imperial College London and the polling firm YouGov have partnered to gather global insights on citizens’ perceptions and responses to the COVID-19 pandemic. [15] The project started the first week of April and surveys have now been systematically conducted in 29 different countries–for a discussion on timing, see the robustness check section. I combined all the individual (country) datasets that include a nationally representative sample of each population. I thus excluded China and India which do not fit this criterion, that is, their samples were not nationally representative. The following countries are thus included: Australia, Brazil, Canada, Denmark, Finland, France, Germany, Hong Kong, Italy, Japan, Malaysia, South Korea, Mexico, Netherlands, Norway, Philippines, Saudi Arabia, Singapore, South Korea, Spain, Sweden, Taiwan, Thailand, United Arab Emirates (UAE), United Kingdom (UK), United States (USA) and Vietnam. While not exhaustive, this sample of countries provides a great deal of variance in terms of many important features such as quality of democracy, economic development and inequality, cultural diversity, political institutions, etc. It is, to my knowledge, the most extensive comparative, individual-level and COVID-19 related dataset.

Overall, it includes a total of 72,417 respondents across 27 countries, with an average age of 45 years old (a standard deviation of 15) and 51% of respondents being women. As mentioned, the samples are nationally representative of a country (based on age, sex, and region). S1 and S2 Tables provides more information on the dataset and the variables. Finally, see the SM’s first page on how to access the replication files.

### Indicators

The surveys systematically included measures of attitudes and self-reported behaviors related to the COVID-19. The two attitudes were (i) prospective self-isolation, and (ii) willingness to isolate. The correlation among the two does not cause concern (Pearson’s r of .31). The question wording and the answer choices are detailed below.

*Prospective self-isolation*: Thinking about the next 7 days … would you isolate yourself after feeling unwell or having any of the following new symptoms: a dry cough, fever, loss of sense of smell, loss of sense of taste, shortness of breath or difficulty breathing? [Yes, No, Not sure]

*Willingness to isolate*: If you were advised to do so by a healthcare professional or public health authority to what extent are you willing or not to self-isolate for 7 days? [Very willing, Somewhat willing, Neither willing nor unwilling, Somewhat unwilling, Very unwilling, Not sure]

Prospective self-isolation is used as a dichotomous variable where ‘yes’ is coded as 1 and ‘no’ (or ‘not sure’) as 0. The willingness to isolate is rescaled from 0 to 1 where 1 corresponds to ‘very willing’ and ‘not sure’ is coded as 0.5, i.e. the same value as ‘neither willing nor unwilling’. To measure self-reported behaviors, we rely on a question asking about the frequency of several preventive measures. Self-reported behavior regarding preventive measures will certainly entail a social desirability bias [16] That said, it would prevent inference *only if* there is systematic bias regarding who is affected by the social desirability bias. We do not have any theoretical reasons to believe so—this is confirmed by Larsen et al. [17] who made use of an experimental design to tackle this issue. In other words, the bias could ideally be reduced and let citizens more readily admit non-compliance, but it should not prevent statistical inference. The question was formulated as follows, with the different items shown below:

“Thinking about the last 7 days… how often have you taken the following measures to protect yourself or others from coronavirus (COVID-19)?

- Worn a face mask outside your home (e.g. when on public transport, going to a supermarket, going to a main road)
- Washed hands with soap and water
- Used hand sanitiser
- Covered your nose and mouth when sneezing or coughing
- Avoided contact with people who have symptoms or you think may have been exposed to the coronavirus
- Avoided going out in general
- Avoided taking public transport
- Avoided having guests to your home
- Avoided small social gatherings (not more than 2 people)
- Avoided medium-sized social gatherings (between 3 and 10 people)
- Avoided large-sized social gatherings (more than 10 people)
- Avoided crowded areas
- Avoided going to shops
- Eaten separately at home, when normally you would eat a meal with others
- Cleaned frequently touched surfaces in the home (e. g. doorknobs, toilets, taps)
- Avoided touching objects in public (e.g. elevator buttons or doors)

I did not include the following four items because they would overwhelmingly apply (or overwhelmingly not apply) to elderly people: (i) Avoided going to hospital or other healthcare settings, (ii) Avoided working outside your home (iii) Avoided letting your children go to school/ university (iv) Slept in separate bedrooms at home, when normally you would share a bedroom–See the first test of the robustness checks section for details.

Answer choices were “Always, Frequently, Sometimes, Rarely, Not at all.” We rescaled all the variables in a 0 to 1 range, excluding the ‘Not sure’. Descriptive statistics for every variable are shown in S2 Table. However, I combined all the 16 items listed above to generate an index of compliance with preventive measures (Cronbach’s α = .86). For the sake of parsimony, I discuss some particular items that prove to be outliers in the findings (see the robustness check section for a discussion on the operationalization of the items).

**Fig 2:**
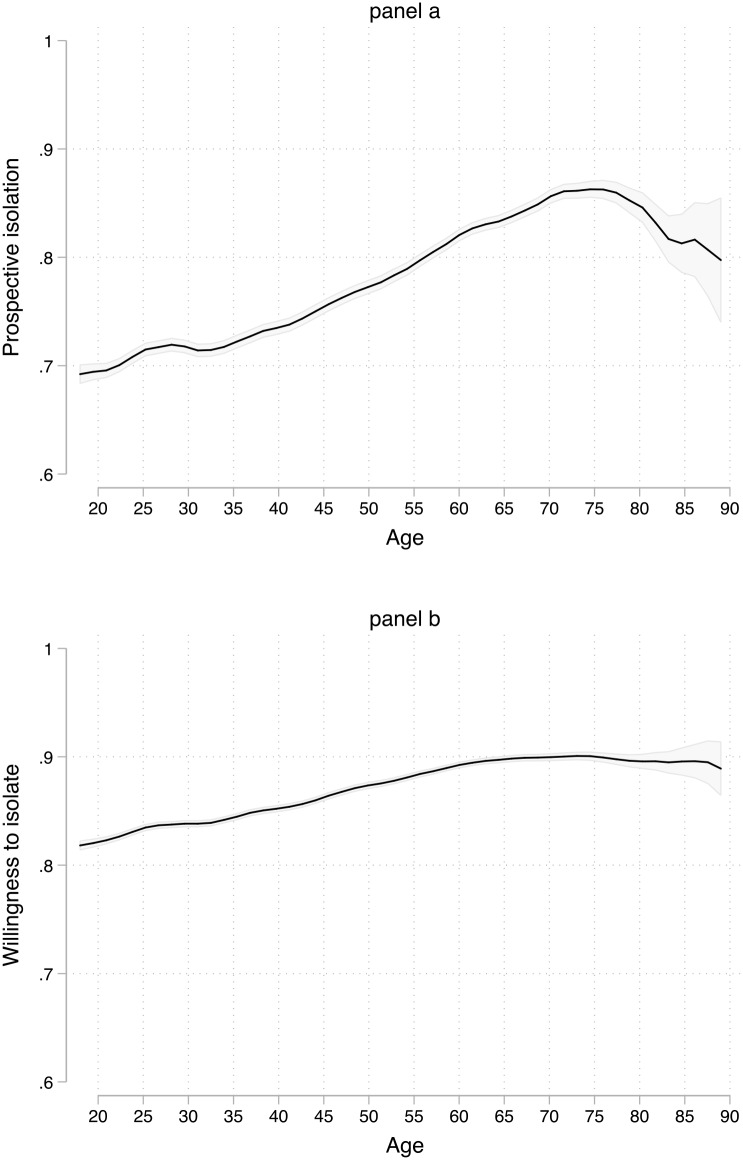
Number of observations and age.

Finally, age ranges from 18 to 90. 14 respondents were in their 90’s, but I excluded age values for which there was less than 10 observations, hence the range 18–90. Fig 2 below shows the distribution of age. Beyond the scope of the dataset, one of its main advantages is that the number of observations allow us to study many age values. There are even several hundreds of respondents over 60 years of age. I do not aim to provide a definition of ‘elderly people’ for the simple reason that, of course, it is a socially constructed concept and can vary among individuals and across countries. Are findings also do not defer whether we consider an elderly to be 60, 65, or 70.

That said, the number of people that are 80 years old or older is quite limited. S3 Table shows the number of observations using 10-year categories and we can see that there are only 544 respondents within the 80–90 years old range. As in several inquiries in social sciences, it is likely that the dataset used in this research does not include a homogenous group of 80+ year-olds due to the lower number of observations in that category. For an example, see Blais and Daoust [18] who mention that they are less confident about their subsample of 75+ year-old respondents. This should be kept in mind and explains the focus on the 18–80 years old range.

## Findings

Considering our main interest, one can access the distribution of age and the measures of attitudes and compliance towards COVID-19 preventive measures in S1 Fig. The bivariate relationships of age with the prospective self- isolation and the willingness to do so are shown in Fig 2. To allow for non-linearity, I used local regressions with the kernel (epanechnikov) function and a bandwidth of 0.8 –see the robustness checks section for a discussion of this choice.

In both cases, using age as a *linear* predictor of these two attitudes toward COVID-19 preventive measures would indicate a strong (panel a) or somewhat important (panel b) relationships. However, this interpretation would be misleading for the simple reason that the relationship is not linear. In panel a, the relationship is flat between 70 and 75 years of age, and declines until the age of 90. For the reasons mentioned above, we should be careful with the 80-90-year-olds even if the confidence intervals increase as the number of observations diminishes. However, it still leaves us with a quite pessimistic interpretation: in the most optimistic case, elderly people would not be substantially more likely to isolate compared to their fellow citizens in their 50’s and 60’s.

In panel b, on the willingness to isolate, the effect of age is much smaller, but clearly positive until 60 years of age. From that point on, there is substantially no relationship. For both attitudes, we clearly fail to see an increased (prospective) disciplined or willingness for people after 70 years old (panel a) and 60 years of age (panel b). This is surprising and quite troubling given the governments’ strategy to publicize the fact that elderly people are much more vulnerable and more likely to need intense care or die from COVID-19. I now turn to compliance with preventive measures.

**Fig 3:**
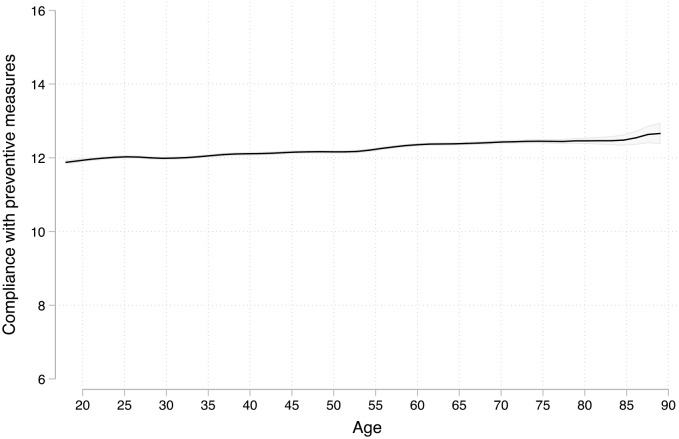
Compliance with preventive measures and age. Local regression with a kernel (epanechnikov) function and a bandwidth of 0.8, with 84% confidence intervals included. [19].

As it is clear from eyeballing Fig 3, this is *no* substantial effect of age on one’s score of compliance with COVID-19 preventive measures. Overall, the score for every age is substantively similar and is around 12. It ranges from a minimum of 11.9 to a maximum of 12.4, for a total effect of 0.5 on a 0 to 16 scale. Put differently, it represents less than a fifth of the standard deviation of the index. There are thus two main conclusions to Fig 3. On the one hand, the baseline level of compliance with preventive measures is quite high, although not overwhelmingly high (especially given that the maximum is 16). On the other hand, there is no substantial effect of age.

The index included 16 very different items. In S2 Fig, I replicated the local regression for each item. Overall, the main conclusion remains the same, there is no substantial effect of age on compliance to COVID-19 public health measures. However, there are some interesting variances. By far the most interesting concerns wearing a mask outside the home.

**Fig 4:**
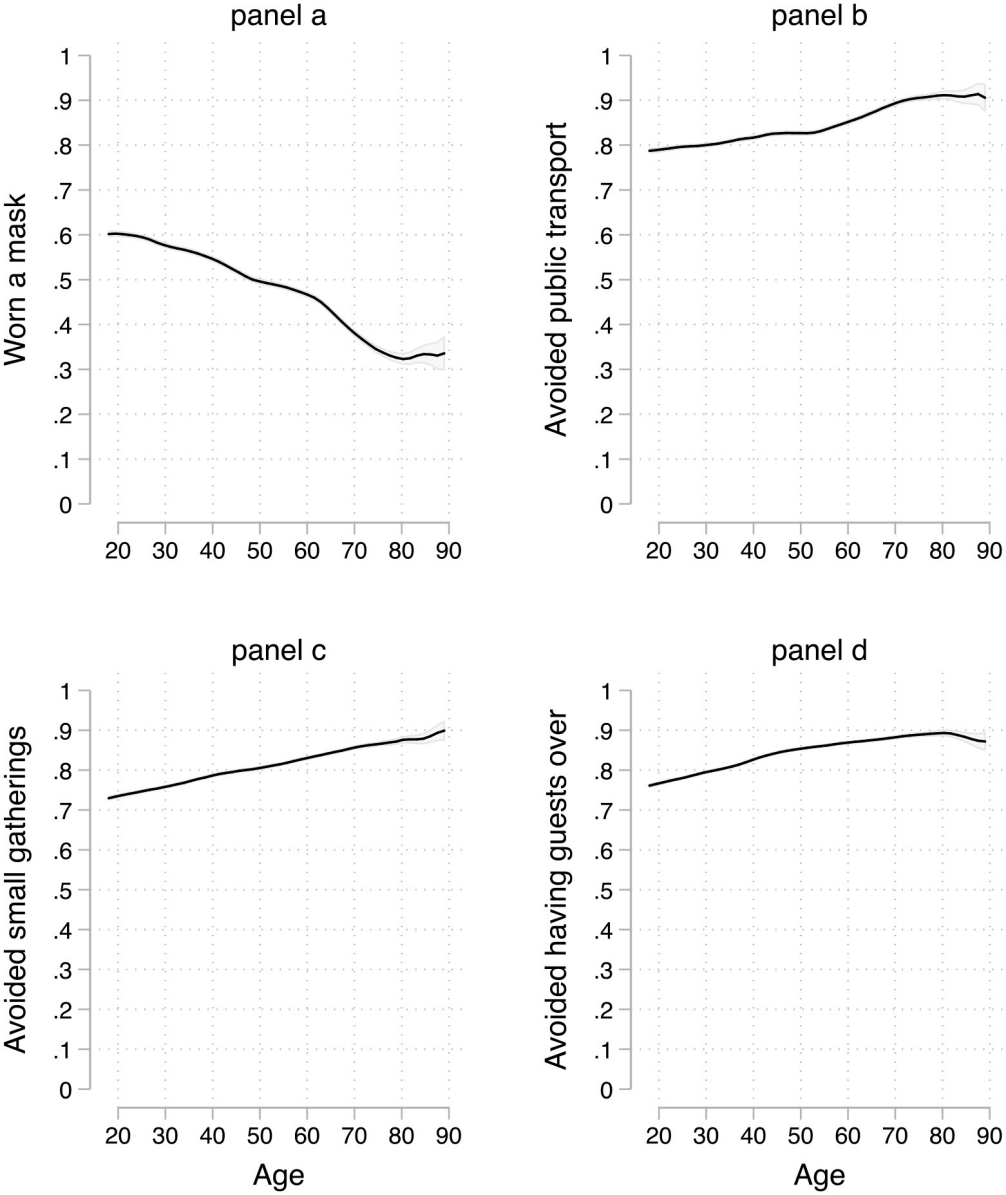
The effect of age on particular preventive measures. Local regression with a kernel (epanechnikov) function and a bandwidth of 0.8, with 84% confidence intervals included. [19].

Fig 4 (panel a) shows the results. The effect of age on wearing a mask outside is negative, quite linear and the strongest among all the relationships for individual items. From 20 to 60 years old, there is a decrease of about .15, and there is another decrease of the same magnitude from 60 to 80 years of age, before stabilizing. The total effect is thus of .3 on a 0 to 1 scale. It is astonishing that this particular age group (60–80 years old), which is much more likely to die from the disease than their younger fellow citizens, comply to a lesser extent with the preventive measure of wearing a mask—see Feng et al. (2020) for a discussion on recommendations around the world regarding face masks in community settings.

Slightly reassuring, the other three items for which the effect is noteworthy (although not even close to the negative impact of age on wearing a mask outside the home) are positive. Older people are more likely to have avoided public transportation, small gatherings, and having guests over. These effects are of a little more than .10.

## Robustness checks

In this section, I test whether different choices in my approach would lead to different conclusions. First, I have excluded four items out of twenty in order to generate the index of compliance (see footnote 3) but including them does not alter the findings. As shown in S3 Fig, there is no substantial relationship. Second, I have coded the ‘Not sure’ as people who would *not* self-isolate if they were told do so for the prospective isolation measure and I have put them at 0.5 (with the ‘neither willing nor unwilling) for the compliance items. S4a and S4b Fig shows that excluding the ‘not sure’ instead of putting them at 0 in the former case and excluding them instead of coding them at the neutral point in the latter case does not change the results.

Second, I opted for an elegant way of presenting the results and did not include covariates to keep other factors constant. Some might worry about the impact of the national context and time (over the five weeks). It is likely that respondents’ attitudes are shaped by what happens in their country, but I cannot think of convincing mobilizing national factors (or time) that would affect the *relationship* (and not the distributions) of age and attitudes toward COVID-19 and compliance to preventive measures. That said, I replicated the models for the main results using (logistic and OLS) regressions and used age as a categorical variable with values ranging from 18 to 90 to allow for non-linear effects. I then estimated the predicted probability to self-isolate for every two-years change (given the number of observations among respondents in their 80’s). That strategy allows to include country fixed-effect and covariates for time and individual-level variables. The dataset does not include the exact date of the interview but as shown in S1 Table, the dates of the field are included. Hence, I included dichotomous variables capturing whether the survey was fielded the first two weeks of April, the last two weeks of April or in the first week of May. For individual-level variable, I include gender, employment status (in 8 categories), having children at home and living alone. [20] S5 Fig shows the results with country fixed-effects, covariates for time and individual-level controls. The patterns are substantially similar.

However, one may also want to include region fixed-effects to adopt a more conservative approach in terms of within-country (regional) differences. The 27 countries vary in terms of how many ‘regions’ they have, but YouGov provides a variable indicating respondent’s region based on what is used for the regional quotas (it can be state, Länder, region, province, etc.). There are on average 8–9 regions per country. S6 Fig replicates the previous Figure with the only exception that I added 236 region fixed effects. This kind of estimation strategy controls for the fact that people in the UK are in the UK as opposed to another country. However, it also controls for a respondent in, for example, Wales (a region of the UK), for whom the reality related to COVID-19 might be different compared to respondents in other regions in the UK (such as Scotland or England, for example). As can be seen, this does not alter the substantial interpretation of the findings. One could think about adding covariates like 236 dummies to capture every region (minus one as a reference category) in the 27 countries included. Also, mixed-effects logistic and linear regressions could be used (where individuals are nested within countries–or within regions, then within countries). All these approaches do not alter the main findings.

Third, I showed local regressions with the default bandwidth of 0.8. S7 and S8 Figs. replicate the findings with a bandwidth of 0.7 and 0.9. These results not only confirm my conclusions but suggest an even more pessimistic scenario. That is, for attitudes about prospective self-isolation and willingness to isolate, the lower confidence intervals decrease among the oldest to a level that is similar to the youngest (people in their 20’s). All in all, the difference is somewhat modest but points toward an even more negative picture.

## Discussion

Governments around the world are working to reduce the number of deaths caused by the COVID-19. While overcoming this pandemic relies on an efficient strategy that involves the whole population, the elderly people are disproportionately affected by this disease. Thus, attitudes toward COVID-19 and compliance toward preventive measures among the older citizens will have a greater effect on minimizing the number of deaths. Hence, there is a burgeoning literature on age and COVID-19, which will be crucial in improving our responses to the pandemic. However, we have no systematic comparative perspective and most importantly age has been conceived to have a linear impact on a variety of dependent variables related to COVID-19. I am not blaming scholars, as even early rigorous work has shown results consistent with this view, in the US but not only. [7, 13] That said, I provided a thorough analysis of the impact of age, with a focus on elderly people, allowing for non-linear effects. Moreover, I did so using a comparative perspective including 27 countries.

The findings show that the elderly people, i.e. the most vulnerable population, are not systematically more responsive in terms of prospective self-isolation (if they were told to do so) and willingness to isolate. Moreover, they are not more disciplined in terms of compliance with preventive measures, especially with wearing a face mask when outside their home. This behaviour will become especially important when social distancing rules will be loosened. This is surprising because it is very reasonable to expect that those who are more likely to be hospitalized and/or die from the COVID-19 will be more disciplined and dutiful. Whether we focus on psychological reactions such as fear or rational calculus, we always have the same expectation. Not only is this surprising but it is also troubling. Why do we find such results? Clearly, it is not due to a ceiling effect. There is room for improvement in every Figure shown in this research, and especially when dealing with compliance (such as the index or wearing a face mask). There may be short-term or more deeply rooted predispositions among older people, that can partly explain this non-effect, but either way, governments must revisit their approach in order to minimize the number of deaths caused by COVID-19.

Methodologically speaking, this research is also important for scholars studying COVID-19. Some have put effort to capture a non-linear effect, but this is not the case for the majority of the work. In particular, while using a US sample and focusing on social distancing, Canning et al. [14] mention: “Holding other factors constant, people over 50 years of age have less than half the expected number of close contacts than people age 18–29.” This sounds like good news, but we know that older people especially after 60 are more affected. Hence, we should not see a positive effect of age until 50, but rather until 70, and ideally more.

All in all, focusing on age is not a panacea, but it disproportionately helps to minimize the number of deaths. [21–22] By contributing to the research on age and the COVID-19, I hope to improve our response, which will lead us closer to that objective.

## Supporting information

S1 Table Country, waves, and number of observations included in the dataset. (DOCX)

S2 Table Descriptive statistics. (DOCX)

S3 Table Age and number of observations. (DOCX)

S1 Fig Distributions of the dependent variables. (DOCX)

S2 Fig Replication Fig 3 with the 16 items separately. (DOCX)

S3 Fig Using all 20 items for the index of compliance with preventive measures. (DOCX)

S4 Fig Alternative operationalization of neutral responses. (DOCX)

S5 Fig The effect of age, with country-fixed and covariates for time and individual-level variables. (DOCX)

S6 Fig The effect of age, with country and region fixed-effects, covariates for time and individual-level variables. (DOCX)

S7 Fig Using local regressions with bandwidth = 0.7. (DOCX)

S8 Fig Using local regressions with bandwidth = 0.9. (DOCX)

S1 File (DO)

S2 File (DO)

S1 Data (ZIP)
