# Supplementary material for: Elderly people and responses to COVID-19 in 27 Countries
Source: PLoS One. 2020 Jul 2;15(7):e0235590. doi: 10.1371/journal.pone.0235590 (PMC7332014; doi:10.1371/journal.pone.0235590)
Supplement: S1 Table — (DOCX) [file pone.0235590.s001.docx]

Table SM.1. Country, waves, and number of observations included in the dataset

| Country | *Wave (dates; N)* | Country | *Wave (dates; N)* |
| --- | --- | --- | --- |
| Australia | 1 (01/04-03/04; 973)  2 (07/04-09/04; 1,005)  3 (15/04-20/04; 1,002)  4 (21/04;24/04; 1,004);  5 (30/04-01/05; 1,006) | Norway | 1 (09/04-14/04; 1007)  2 (30/04-01/05; 1,005) |
| Brazil | 1 (02/04-08/04; 895) | Philippines | 1 (08/04-13/04; 993)  2 (30/04-01/05; 968) |
| Canada | 1 (02/04-06/04; 1,003)  2 (30/04-01/05; 1,001) | Saudi Arabia | 1 (08/04-16/04; 940) |
| Denmark | 1 (09/04-12/04; 1,005)  2 (28/04-30/04; 1,008) | Singapore | 1 (03/04-07/04; 1,007)  2 (07/04-13/04; 1,002) |
| Finland | 1 (08/04-13/04; 1,008)  2(27/04-30/04; 999) | South Korea | 1 (02/04-09/04; 938) |
| France | 1 (02/04-03/04; 1,000)  2 (07/04-09/04; 1,001)  3 (15/04-17/04; 1,010)  4 (24/04-27/04; 1,002)  5 (30/04-03/05; 1,002) | Spain | 1 (02/04-03/04; 1,005)  2 (07/04-09/04; 1,010)  3 (09/04-15/04; 1,005)  4 (24/04-27/04; 986)  5 (30/04-01/05; 1,003 |
| Germany | 1 (02/04-03/04; 1,001)  2 (07/04-13/04; 1,012)  3 (15/04-17/04; 1,001)  4 (24-04-27/04; 1,003)  5 (30/04-01/05; 1,001) | Sweden | 1 (03/04-05/04; 1,001)  2(07/04-14/04; 1,010)  3 (16/04-20/04; 1,009)  4 (24/04-29/04; 1,012)  5 (30/04-01/05; 1,000) |
| Hong Kong | 1 (09/04-16/04; 928) | Taiwan | 1 (08/04-13/04; 1,007)  2 (27/03-30/04; 980) |
| Indonesia | 1 (08/04-12/04; 1,018)  2 (30/04-01/05; 1,008) | Thailand | 1 (09/03-13/04; 1,007)  2 (27/04-29/04; 1,016) |
| Italy | 1 (02/04-04/04; 999)  2 (07/04-09/04; 1,004)  3 (15/04-17/04; 1,000)  4 (24/04-27/04; 1,000)  5 (30/04-01/05; 1,005) | United Arab Emerites | 1 (09/04-12/04; 967) |
| Japan | 1 (02/04-08/04; 997) | United Kingdom | 1 (01/04-02/04; 1,649)  2 (07/04-09/04; 1,000)  3 (15/04-21/04; 1,002)  4 (21/04-24/04; 999)  5 (30/04-01/05; 1,004) |
| Malaysia | 1 (09/04-13/04; 1,016)  2 (30/04-01/05; 1,007) | United States | 1 (02/04-07/04; 2,008)  2 (07/04-14/04; 1,005)  3 (15/04-20/04; 1,013)  4 (21/04-30/04; 948) |
| Mexico | 1 (03/04-06/04; 1,004)  2 (27/04-30/04; 986) | Vietnam | 1 (09/04-13/04; 1,003)  2 (30/04-01/05; 1,006) |
| Netherlands | 1 (09/04-14/04; 982)  2 (30/04-02/05; 1,002) |  |  |

Note: Total number of observations is 72,417
