# Supplementary material for: Elderly people and responses to COVID-19 in 27 Countries
Source: PLoS One. 2020 Jul 2;15(7):e0235590. doi: 10.1371/journal.pone.0235590 (PMC7332014; doi:10.1371/journal.pone.0235590)
Supplement: S2 Table — (DOCX) [file pone.0235590.s002.docx]

Table SM.2. Descriptive statistics

|  | Min | Mean | Std. Dev. | Max | Number of obs. |
| --- | --- | --- | --- | --- | --- |
| Age | 18 | 44.809 | 16.552 | 89 | 72417 |
| Sex (Woman=1) | 0 | 0.509 | 0.500 | 1 | 72417 |
| Having children | 0 | 0.441 | 0.497 | 1 | 71586 |
| Live alone | 0 | 0.155 | 0.362 | 1 | 66070 |
| *Employment status* |  |  |  |  |  |
| Full time employment | 0 | 0.428 | 0.495 | 1 | 57352 |
| Full time student | 0 | 0.065 | 0.247 | 1 | 57352 |
| Not working | 0 | 0.083 | 0.276 | 1 | 57352 |
| Other | 0 | 0.033 | 0.178 | 1 | 57352 |
| Part time employment | 0 | 0.136 | 0.343 | 1 | 57352 |
| Retired | 0 | 0.156 | 0.363 | 1 | 57352 |
| Unemployed | 0 | 0.100 | 0.300 | 1 | 57352 |
| *Timeline* |  |  |  |  |  |
| April 1-15 | 0 | 0.491 | 0.500 | 1 | 72417 |
| April 16-30 | 0 | 0.316 | 0.465 | 1 | 72417 |
| May 1^st^+ | 0 | 0.194 | 0.395 | 1 | 72417 |

*Note: For other descriptive statistics, see Figure 1, SM1 and SM2.*
