# Supplementary material for: Elderly people and responses to COVID-19 in 27 Countries
Source: PLoS One. 2020 Jul 2;15(7):e0235590. doi: 10.1371/journal.pone.0235590 (PMC7332014; doi:10.1371/journal.pone.0235590)
Supplement: S3 Table — (DOCX) [file pone.0235590.s003.docx]

Table SM.3. Age and number of observations

| Age group | Number of observations | Percentage (%) |
| --- | --- | --- |
| 18-29 | 16,648 | 23 |
| 30-39 | 14,358 | 19.8 |
| 40-49 | 12,409 | 17.1 |
| 50-59 | 12,264 | 16.9 |
| 60-69 | 10,911 | 15.1 |
| 70-79 | 5,283 | 7.3 |
| 80-89 | 544 | 0.8 |

*Note: Total number of observations is 72,417*
