# Supplementary material for: Elderly people and responses to COVID-19 in 27 Countries
Source: PLoS One. 2020 Jul 2;15(7):e0235590. doi: 10.1371/journal.pone.0235590 (PMC7332014; doi:10.1371/journal.pone.0235590)
Supplement: S1 Fig — (DOCX) [file pone.0235590.s004.docx]

Figure SM.1. Distributions of the dependent variables

*Note: Total number of observations is 72,417*
