# Supplementary material for: Elderly people and responses to COVID-19 in 27 Countries
Source: PLoS One. 2020 Jul 2;15(7):e0235590. doi: 10.1371/journal.pone.0235590 (PMC7332014; doi:10.1371/journal.pone.0235590)
Supplement: S5 Fig — (DOCX) [file pone.0235590.s008.docx]

Figure SM.5. The effect of age, with country-fixed and covariates for time and individual-level variables

*Notes: Estimations from logistic (panel a) and OLS (panel b and c) regression with country fixed-effects, covariates for time (distinguishing the five weeks in early April, end of April, and early May) and individual-level controls (gender, having children, employment status – using 8 categories). See the replication files and the codebook, publicly available, for more information.*
