# Supplementary material for: Elderly people and responses to COVID-19 in 27 Countries
Source: PLoS One. 2020 Jul 2;15(7):e0235590. doi: 10.1371/journal.pone.0235590 (PMC7332014; doi:10.1371/journal.pone.0235590)
Supplement: S7 Fig — (DOCX) [file pone.0235590.s010.docx]

Figure SM.7. Using local regressions with bandwidth=0.7

*Note: local regression with a kernel (epanechnikov) function and a bandwidth of 0.8, with 84% confidence intervals included.*^[19]^
